# Intermittent and graded exercise effects on NK cell degranulation markers LAMP‐1/LAMP‐2 and CD8^+^ CD38^+^ in chronic fatigue syndrome/myalgic encephalomyelitis

**DOI:** 10.14814/phy2.13091

**Published:** 2017-03-08

**Authors:** Suzanne Broadbent, Rosanne Coutts

**Affiliations:** ^1^ School of Health and Human SciencesSouthern Cross UniversityLismoreAustralia

**Keywords:** Chronic fatigue, graded exercise therapy, intermittent training, natural killer cells, t‐cytotoxic lymphocytes

## Abstract

There is substantial evidence of immune system dysfunction in chronic fatigue syndrome/myalgic encephalomyelitis (CFS/ME) but little is understood of exercise training effects on lymphocyte function in this illness. This study investigated whether graded and intermittent exercise improved CD8^+^ lymphocyte activation and natural killer cell degranulation markers compared to no exercise. Twenty‐four chronic fatigue syndrome (CFS) patients (50.2 ± 10 year) were randomized to graded exercise (GE), intermittent exercise (IE) or usual care (UC) groups; a control group (CTL) of 18 matched sedentary non‐CFS/ME participants were included for immunological variable comparisons. Main outcome measures were pre‐ and postintervention expression of CD3^+^ CD8^+^ CD38^+^ and CD3^−^ CD16^+^56^+^ CD107a^+^ (LAMP‐1) CD107b^+^ (LAMP‐2) and aerobic exercise capacity. The postintervention percentage of NK cells expressing LAMP‐1 and ‐2 was significantly higher in IE compared to UC, and higher in GE compared to UC and CTL. LAMP‐1 and LAMP‐2 expression (absolute numbers and percent positive) increased significantly pre‐to‐postintervention for both GE and IE. Preintervention, the absolute number of CD8^+^ CD38^+^ cells was significantly lower in CTL compared to UC and IE. There were no significant pre‐ to postintervention changes in CD8^+^ CD38^+^ expression for any group. Aerobic exercise capacity was significantly improved by GE and IE. Twelve weeks of GE and IE increased the expression of NK cell activation and degranulation markers, suggesting enhanced immunosurveillance. Low‐intensity exercise may also reduce CD8^+^ CD38^+^ expression, a marker of inflammation. Both GE and IE improved exercise capacity without worsening CFS/ME symptoms, and more robust trials of these exercise modalities are warranted.

## Introduction

Chronic fatigue syndrome/myalgic encephalomyelitis (CFS/ME) is a debilitating disorder with possible multifactorial origins. Suspected triggers include severe viral infections and emotional stress (Lorusso et al. 2009). The illness is characterized by postexertional malaise and severe, unexplained fatigue, usually combined with myalgia, flu‐like symptoms, swollen lymph nodes and recurring infections (Carruthers et al. 2011). There is considerable evidence of widespread immune dysfunction and elevated inflammatory cytokines in CFS/ME patients, but there has been variation in the outcome measures and magnitude of these studies. Many investigations found equivocal results of the numbers and function of leukocyte subsets and cytokine concentrations (Lorusso et al. 2009; Nijs et al. 2014). However, there are consistent reports of a decrease in natural killer (NK) cell cytotoxic activity (Brenu et al. 2012, 2013; Nijs et al. 2014). Cytotoxic lymphocyte CD3^+^CD8^+^ numbers and function in CFS/ME patients are less clear. CD3^+^CD8^+^ concentrations may be increased in CFS patients (Lorusso et al. 2009), or no different from non‐CFS (Brenu et al. 2010; Cohnen et al. 2013; Lorusso et al. 2009). Maher et al. (2005) reported that both NK and CD8^+^ lymphocytes showed lower levels of intracellular perforin compared to non‐CFS/ME. This reduced cytotoxic capacity may be associated with a diminished immune surveillance, but whether it is a contributing factor to the development of CFS/ME or results from the illness has not been determined.

NK cells are granular lymphocytes found in peripheral blood, bone marrow, and lymphoid tissues (Schenk et al. 2016). The cells are defined by the surface markers CD56 and CD16, in the absence of T‐cell marker CD3; the CD56^bright^CD16^+^ subgroup has a lower cytotoxic capacity but greater release of cytokines such as interferon (IFN)‐*γ* and tumor necrosis factor (TNF)‐*α*, whereas the CD56^dim^CD16^+^ subgroup, which constitute about 90% of circulating NK cells, display high cytotoxic activity (Schenk et al. 2016). NK cells can directly lyse tumor or virus‐infected cells without prior stimulation or direct exposure to a specific antigen (Arnon et al. 2006). This activity appears early in the immune response, providing protection during the time needed for the activation and proliferation of cytotoxic T lymphocytes, and their differentiation into functional cells (Koch et al. 2013). The lysing ability of both NK and CD3^+^CD8^+^ cells depends upon the secretion of lytic granules (e.g., perforin, lysosome‐associated membrane proteins CD107a/LAMP‐1 and CD107b/LAMP‐2) at the immunologic synapse with the target cells (Aktas et al. 2009; Cohnen et al. 2013; Krzewska et al. 2013; Tomescu et al. 2009). The expression of LAMP‐1 on CD8^+^ lymphocytes has been regarded as a sensitive activation marker for some time (Alter et al. 2004) but only relatively recently have studies found that both LAMP‐1 and LAMP‐2 are equally sensitive as a degranulation markers in NK cells (Aktas et al. 2009; Krzewska et al. 2013; Tomescu et al. 2009). Recent studies reported that (1) intracellular expression of LAMP‐1 and LAMP‐2 positively correlated with cytotoxic granule content; (2) LAMP‐1 and LAMP‐2 protected NK cells during the degranulation process, avoiding NK apoptosis (Cohnen et al. 2013); and (3) reduced LAMP‐1 expression inhibited the movement of lytic granules and decreased perforin levels (Krzewska et al. 2013). Therefore, the expression of both LAMP‐1 and LAMP‐2 on NK cells in CFS/ME patients may be an indicator of the cytotoxic capacity of the cells, and the immune status of the patients.

Cytotoxic T cells (CD3^+^CD8^+^) are vital for recognition and clearance of bacteria‐infected cells (Strioga et al. 2011). They recognize their targets by binding to antigen associated with MHC class I molecules, which co‐stimulates the CD28 receptor; once activated they release perforin and granzymes (Strioga et al. 2011). They can also express the surface protein FAS ligand (FasL), which assist in initiating apoptosis. Some studies found that the CD3^+^CD8^+^ cytotoxic function in CFS/ME patients was significantly less than non‐CFS/ME controls, suggesting impaired lymphocyte function (Brenu et al. 2010; Curriu et al. 2013; Hardcastle et al. 2015). Curriu et al. (2013)found a lower frequency of effector memory cells, lower CD38^+^ expression, and higher numbers of anergic lymphocytes (expressing CD5) compared to non‐CFS/ME. However, Robertson et al. (2005) reported higher concentrations of CD8^+^ effector cells, CD38 and HLA DR activation, suggesting a chronically activated immune response or low‐level systemic inflammation.

CD38 has been referred to as an “activation marker” for CD3^+^CD8^+^ lymphocytes but it is part of the gene family that codes for bidirectional cellular signaling, and is currently considered to be a multifunctional ectoenzyme as well as a receptor (Quarona et al. 2013). CD38 signaling helps regulate T‐cell activation and proliferation; produces IL‐1, TNF‐*α*, and granulocyte‐macrophage colony‐stimulating factor (GM‐CSF); and when interacting with adhesion molecule CD31, initiates a signaling cascade and leukocyte migration (Almeida et al. 2002; Quarona et al. 2013). The enzymatic functions of CD38 involve the conversion of nicotinamide adenine dinucleotide (NAD^+^) into nicotinamide, adenosine diphosphate‐ribose (ADPR), and cyclic ADPR (Hamblin 2003). By regulating intracellular levels of NAD^+^ and calcium, CD38 helps modulate cell protection, DNA repair, and apoptosis (Quarona et al. 2013). Elevated CD8^+^CD38^+^ is associated with the progression of chronic lymphocytic leukemia, multiple myeloma, human immunodeficiency virus (HIV) (Almeida et al. 2002; Quarona et al. 2013), and Epstein–Barr virus (EBV) (Almeida et al. 2002; Zidovec Lepej et al. 2003). Although EBV is considered a possible cause of CFS/ME (Lorusso et al. 2009), it is unclear whether high or low expression of CD38^+^ is involved with the progression of CFS/ME, and even less clear if exercise has any effect on the expression of this receptor.

Currently, there is no cure for CFS/ME but recognized treatments include rest, specialist medical care, cognitive behavioral therapy (CBT), and graded (self‐paced) exercise therapy (GET) (Carruthers et al. 2011; Wallman et al. 2004). Some studies have reported positive outcomes for GET, with and without CBT (Gordon et al. 2010; Larun et al. 2015; Wallman et al. 2004) but not all participants reported improvements in symptoms or exercise capacity. While too frequent or vigorous exercise may worsen CFS/ME symptoms, little is known how the immune system of CFS/ME patients responds to regular physical activity/exercise (Nijs et al. 2014). Most exercise‐related CFS/ME immunological studies have investigated leukocyte and lymphocyte trafficking, and cytokine levels after an acute bout of exercise (for review, see (Nijs et al. 2014), reporting transient leucocytosis. In healthy individuals, both low and high intensity bouts of exercise increase mobilization of CD3^+^CD8^+^ and NK lymphocytes in peripheral circulation (Campbell et al. 2016). Although graded exercise (GE) effects on cytotoxic cells have not been investigated, a recent study of breast cancer patients undergoing acute intermittent exercise (IE) reported that NK cells were mobilized to the same extent as in healthy women, and that cytolytic activity remained normal, suggesting that IE could confer benefits in terms of maintaining NK cell function (Evans et al. 2015).

We previously published the effects of both GE and IE on CD4^+^ (T‐helper) lymphocyte function, showing that both exercise modalities enhanced CD3^+^CD4^+^ function in CFS/ME patients (Broadbent and Coutts 2016). To understand the effects of exercise modalities on cytotoxic lymphocyte function in individuals with CFS/ME, this 12‐week pilot trial focused on NK cell degranulation markers LAMP‐1/LAMP‐2 and CD8^+^CD38^+^ expression following GE and IE, compared to normal care.

## Methods

### Study design

This pilot study was a 12‐week randomized controlled trial. CFS/ME participants were allocated to an IE or GE cycling group or to usual care (UC, rest and lifestyle advice). Primary outcome measures were full blood count indices (FBC), circulating leukocyte subset counts, CD4^+^CD25^+^CD134^+^ lymphocyte activation (previously published FBC, leukocyte subset count, and CD3^+^CD4^+^ data are not included in this paper; refer to (Broadbent and Coutts 2016); stimulated T‐cytotoxic lymphocyte (CD3^+^CD8^+^) counts and expression (percent positive) of CD8^+^ and CD38^+^; stimulated natural killer (NK) cell counts (CD3^−^CD16^+^CD56^+^) and expression of CD107a (LAMP‐1) and CD107b (LAMP‐2); and aerobic capacity. A matched control group of sedentary non‐CFS/ME participants (CTL) participated for comparison of full blood count and immune cell analyses only. Heart rate, blood pressure, body weight, and exercise test variables were also assessed (Broadbent and Coutts 2016). All study procedures were approved by Southern Cross University Human Research Ethics Committee (HREC ECN‐13‐066) and the Australia and New Zealand Clinical Trials Registry (ACTRN12612001241820).

### Participants

Study inclusion criteria were a diagnosis of CFS/ME according to the Centres for Disease Control (1994) criteria (Fukuda et al. 1994); an age range of 18 to 65 years; no diagnosed cardiopulmonary, metabolic or endocrine condition or musculoskeletal injury that would prevent exercise participation; being able to communicate in English; and provision of informed consent. CTL group participants were age‐ and gender‐matched to CFS/ME participants. Participants were recruited from the local community through advertisements at Southern Cross University, medical clinics and hospitals, and local media. CFS/ME participants were randomized into the exercise intervention or UC groups as published previously (Broadbent and Coutts 2016). CFS/ME participants in the UC group were offered a 12‐week exercise program after the study had been completed. All study participants completed a full medical history. CFS/ME participants also provided anthropometric measures, resting heart rate and blood pressure, and completed a graded incremental aerobic exercise test. UC participants were asked to follow the advice of their medical practitioner (rest, maintaining activity for daily activities) and to avoid any other physical activity during the study, as were the CTL group.

### Exercise testing

The pre‐ and postintervention incremental exercise test methodology for this research project has been previously published (Broadbent and Coutts 2016). Briefly, each CFS/ME participant completed a pre‐ and postintervention incremental cycling test to their maximal effort (Lode Excalibur, Holland) with peak *V̇*O_2_ measured using open circuit spirometry (Moxus, AOL Technologies, Australia). The preintervention test determined each participant's peak exercise heart rate, *V̇*O_2_, RER, perceived exertion (RPE), and power as a basis for their exercise session intensities. Participants completed a three minute warm up of unloaded cycling with the workload then increasing by 10 W per min until maximal effort was achieved. RPE and fatigue were recorded at rest and during the exercise test.

### Exercise interventions

The 12‐week intervention consisted of either IE or GE, three sessions a week using a spin cycle ergometer (Keiser M3i, USA). The exercise sessions were run at the Southern Cross University fitness center, and were supervised by an accredited Exercise Physiologist and trained postgraduate students, with heart rate, blood pressure, and fatigue levels monitored before and after each exercise session. Each session consisted of a 5‐min warm up of unloaded cycling, followed initially by a 10–15 min block of either GE (50% *V̇*O_2peak_, RPE 3 [Modified Borg Scale, (Borg 1982)] or IE, of 1 min of moderate intensity cycling (60% *V̇*O_2peak_, RPE 4–5) alternated with 1 min of unloaded or very low intensity cycling (30% *V̇*O_2peak_, RPE 1–2). The GE protocol was based on the exercise intensity used by previous GE research (Wallman et al. 2004), while the IE protocol was adapted from similar protocols for pulmonary and heart failure patients who are typically deconditioned, and who fatigue quickly, although the mechanisms of fatigue differs to CFS/ME (Butcher and Jones 2006; Piepoli et al. 2011; Puhan et al. 2006). Recommended cadence was between 50 and 70 RPM. The workloads (W) were determined from each participant's baseline *V̇*O_2peak_ cycle test. Exercise sessions were self‐paced and progressed by increasing exercise session duration as tolerated for each participant; workload was not increased until each participant had achieved three consecutive exercise sessions of 30 min in total with no increase in symptoms. The exercise intensity was reduced if any participant reported a worsening in symptoms.

### Immune cell analyses

Blood collection for FBC, analyses of leukocyte subsets and T‐helper lymphocytes, and study blinding procedures have been described elsewhere (Broadbent and Coutts 2016). All participants were requested to abstain from physical activity for 24 h prior to nonfasting blood draws, which were collected between the hours of 0800 and 1000. For flow cytometric analyses of stimulated NK cell degranulation (CD107a/LAMP‐1 and CD107b/LAMP‐2) and CD8^+^CD38^+^ expression, 6 mL of blood were drawn into a sodium heparin Vacutainer tube (BD Biosciences, Australia). The whole blood assay was adapted from procedures described by Betts et al. (2003)and Alter et al. (2004). Sodium heparin anticoagulated whole blood (250 *μ*L) was mixed with 250 *μ*L RPMI‐1640 (Sigma‐Aldrich, Australia), and stimulated by 20 *μ*L of phorbol‐12‐mysitate‐12‐acetate (PMA) solution diluted to 2.5 *μ*g/mL, and 2.5 *μ*L of ionomycin 20 mmol/L solution (Sigma‐Aldrich, Australia), for 1 h at 37°C in a 5% CO_2_ incubator. At that point, 10 *μ*L of Brefeldin A (Sigma‐Aldrich, Australia) at a concentration of 10 *μ*g/mL was added to each sample, with further incubation for 3–4 h. After culture, 100 *μ*L of sample was stained with the following conjugated fluorochromes for 15 min in the dark at room temperature (CD3‐Per‐CP 10 *μ*L; CD8‐APC‐H7 5 *μ*L; CD38‐PE‐Cy7 5 *μ*L; CD16‐PE 5 *μ*L and CD56‐PE 10 *μ*L; CD107a‐APC 5 *μ*L; CD107b‐FITC 10 *μ*L, BD Biosciences, Australia), followed by lysing with 1 mL of BD FACS lysing solution (BD Biosciences, Australia) diluted 1:10. Samples were vortexed then incubated in the dark for a further 15 min before being washed twice in PBS (2 mL) and centrifuged at 300*g* for 5 min. The supernatant was aspirated and the lymphocytes resuspended in 0.5 mL of BD stabilizing fixative (BD Biosciences, Australia) before vortexing and multi‐parameter flow cytometric analysis using FACSDiva v6.1 software (BD Biosciences, Australia); 50,000 cells were analyzed per sample. The CD3^+^ lymphocyte population was first gated according to forward and side scatter properties. From the total lymphocyte population, CD8^+^ and NK cells were identified, gated, and analyzed for expression of CD38, and LAMP‐1/LAMP‐2, respectively.

### Statistical analysis

Sample size and power were calculated as described previously (Broadbent and Coutts 2016). Primary analysis with intention‐to‐treat included all participants regardless of adherence and attrition, and all data were examined for normal distribution. Physiological and immunological data are shown as mean ± standard deviation. Changes between groups and the time effect for each group were determined by repeated measures ANOVA with a Bonferroni adjustment for significant differences between groups. The alpha level was set at ˂0.05. A modified Cohen's Effect sizes (ES) was also calculated for pre‐ to postintervention changes for a magnitude‐based approach, with the following descriptors: ES thresholds: 0.0–0.2 trivial, 0.2–0.6 small, 0.6–1.2 moderate, 1.2–2.0 large, >2.0 very large (Batterham and Hopkins 2006).

## Results

### Participant characteristics

Preintervention, there were no significant differences between groups with respect to physical and physiological characteristics (Table 1), and there were no significant changes in these outcomes postintervention. The mean ages of the combined CFS/ME participants (*n* = 24) and non‐CFS/ME group (*n* = 18) were 50.9 ± 10 year and 50.6 ± 10 year, respectively. The female‐to‐male ratios for CFS/ME and CTL were 17:7 and 13:5, respectively. Time since diagnosis for CFS/ME participants was 2.9 ± 2.6 year.

**Table 1:** CFS/ME participant characteristics and exercise test results pre‐ and postintervention

Variable	UC (*n* = 8)			GE (*n* = 8)			IE (*n* = 8)		
	Pre	Post	% Δ (ES)	Pre	Post	% Δ (ES)	Pre	Post	% Δ (ES)
Age (year)	50.2 ± 11	—	—	51.0 ± 9	—	—	49.4 ± 9	—	—
Height (cm)	166.4 ± 10	—	—	167.1 ± 5	—	—	166.8 ± 9	—	—
Weight (kg)	78.7 ± 22	79.5 ± 22	1.0 (0.04)	69.0 ± 15	70.2 ± 16	1.7 (0.07)	70.0 ± 16	69.7 ± 17	0.4 (0.01)
Resting HR (bpm)	75.8 ± 13	78.0 ± 13	2.9 (0.2)	77.5 ± 13	73.6 ± 14	−5.0 (0.3)	70.8 ± 11	73.0 ± 12	3.1 (0.2)
Resting SBP (mmHg)	125.7 ± 15	124.8 ± 15	0.7 (0.1)	124.2 ± 12	121.0 ± 11	−2.6 (0.3)	127.1 ± 10	120.1 ± 14	− 5.5 (0.6)
Resting DBP (mmHg)	78.5 ± 11	79.4 ± 11	1.1 (0.1)	79.2 ± 10	75.9 ± 7	−4.2 (0.4)	76.0 ± 5	74.1 ± 9	−2.5 (0.3)
*V*O_2peak_ (mL·kg^−1^·min^−1^)	18.6 ± 7	19.7 ± 8	5.9 (0.1)	20.5 ± 5	23.2 ± 4^a^	13.2 (0.6)	20.3 ± 6	24.5 ± 7^a^	20.7 (0.7)
RER_peak_	1.10 ± 0.1	1.10 ± 0.1	0.0 (0)	1.04 ± 0.1	1.09 ± 0.1	4.6 (0.5)	1.11 ± 0.1	1.14 ± 0.1	2.7 (0.5)
Peak power (W)	92.7 ± 33	94.2 ± 39	1.6 (0.1)	96.2 ± 11	102.5 ± 15	6.2 (0.5)	100.0 ± 14	108.8 ± 12	8.8 (0.6)
*V*_Epeak_ (L·min^−1^)	43.4 ± 15	44.7 ± 14	3.0 (0.1)	44.5 ± 11	52.7 ± 14	18.4 (0.6)	48.5 ± 13	58.4 ± 11^a^	20.4 (0.6)
RPE (0 10)	6.6 ± 1	6.6 ± 1	0.0 (0)	6.7 ± 1	6.9 ± 1	3.0 (0.1)	7.1 ± 1	7.1 ± 1	0.0 (0)
Elapsed test time (min)	10.7 ± 3	11.3 ± 4	5.6 (0.2)	10.8 ± 2	11.9 ± 2	10.2 (0.6)	10.8 ± 3	12.9 ± 3^a^	19.4 (0.6)

Data shown as mean ± SD with % change and effect size (ES).

Postintervention value significantly different to pre‐, *P* ˂ 0.050. CFS/ME, chronic fatigue syndrome/myalgic encephalomyelitis.

### Exercise tests and aerobic capacity

Pre‐ and postintervention exercise tests were conducted with CFS/ME participants only (Table 1). Preintervention, there were no significant differences between groups. Postintervention, *V̇*O_2peak_ increased significantly in both IE and GE groups (*P *=* *0.027 ES: 0.7 and *P *=* *0.048 ES: 0.6, respectively). Elapsed test time (ETT) and *V̇*_Epeak_ increased significantly in the IE group (*P *=* *0.034; ES: 0.6; *P *=* *0.004; ES: 0.6, respectively), with trends to increase in *V̇*_Epeak_ and RER_peak_ for the GE group (*P *=* *0.075 ES: 0.6 and *P *=* *0.060 ES: 0.5, respectively) (Table 1).

### Stimulated CD3^+^CD8^+^CD38^+^ expression

**Figure 1:**
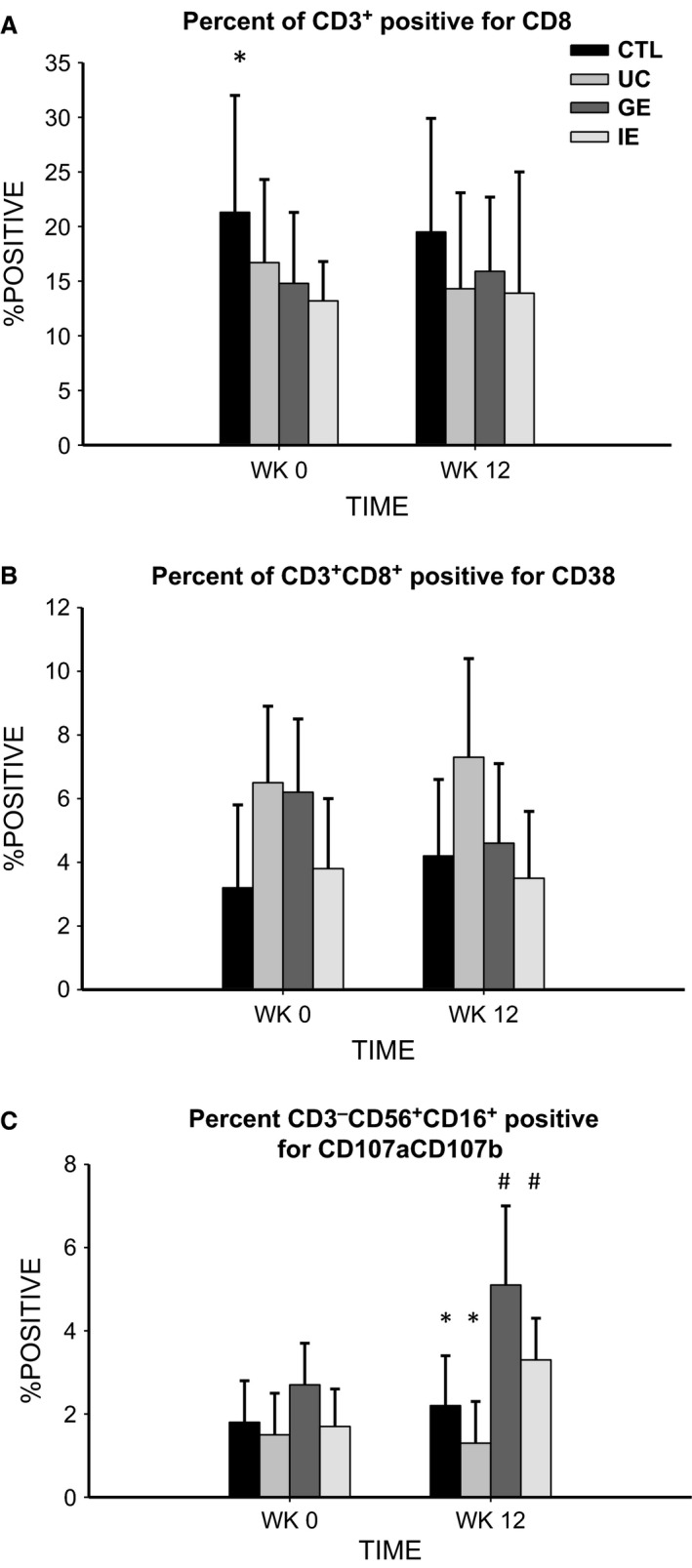
Changes in the percentage positive of stimulated CD3^+^ lymphocytes expressing (A) CD8^+^, (B) CD8^+^ CD38^+^; and (C) of CD3^‐^ CD56^+^ CD16^+^ expressing LAMP‐1 and LAMP‐2 before and after 12 weeks of GE, IE, or UC in CFS/ME individuals compared to non‐CFS/ME controls. *(A) CTL group significantly higher than IE; and (C) CTL and UC significantly lower than GE. ^#^(C) Group postintervention values for GE and IE significantly higher than preintervention values. CFS/ME, chronic fatigue syndrome/myalgic encephalomyelitis; CTL, control group; IE, intermittent exercise.

The absolute numbers and percentage positive of CD3^+^CD8^+^CD38^+^ are shown in Table 2 and Figure 1, respectively. At Week 0, the CTL group had a significantly higher percentage of CD3^+^ lymphocytes positive for CD8 than the IE group (*P *=* *0.030), and showed a trend to being higher than GE (*P *=* *0.070). There were no significant between‐group differences postintervention, nor were there any significant changes over the 12 weeks for any group with absolute numbers or percent positive.

**Table 2:** Stimulated CD3^+^CD8^+^CD38^+^ and CD3^−^CD56^+^CD16^+^LAMP1/2 counts pre‐ and postintervention

Variable	CTL (*n* = 18)			UC (*n* = 8)			GE (*n* = 8)			IE (*n* = 8)		
	Pre	Post	% Δ (ES)	Pre	Post	% Δ (ES)	Pre	Post	% Δ (ES)	Pre	Post	% Δ (ES)
CD3^+^CD8^+^CD38^+^	697.0 ± 442^b^	960.8 ± 631	37.8 (0.1)	1367.2 ± 314	1408.0 ± 205	3.0 (0.1)	1281.6 ± 215	948.5 ± 280	−26.0 (0.7)	1494.0 ± 292	1174.1 ± 212	−21.4 (0.6)
CD3^−^CD16^+^CD56^+^	435.4 ± 201	478.0 ± 184	9.8 (0.1)	477.1 ± 283	525.2 ± 269^c^	10.1 (0.1)	612.0 ± 216	858.4 ± 283^a^	40.3 (0.8)	569.4 ± 204	756.5 ± 203^a^	32.9 (0.6)

Data shown as mean ± SD with % change and effect size (ES); CFS/ME, chronic fatigue syndrome/myalgic encephalomyelitis;

a Pre‐ to postintervention values significantly different for GE and IE, *P *<* *0.050.

b Preintervention value significantly different for CTL compared to UC, GE and IE, *P *<* *0.050.

c Between‐group postintervention value significantly different, *P* ˂ 0.050, for UC compared to GE, graded exercise and IE, intermittent exercise.

At Week 0, the CTL group had significantly lower absolute numbers of CD3^+^CD8^+^ expressing CD38^+^ compared to UC (*P *=* *0.043) and IE (*P *=* *0.022) and showed a trend to significance compared to GE (*P *=* *0.067) (Table 2). There were no between‐group differences at Week 12 and no significant pre‐ to postintervention changes for any group. There were also no differences between groups at either Week 0 or Week 12 for the percentage of CD3^+^CD8^+^ positive for CD38^+^, and there were no significant pre‐ to postintervention changes for any group.

### Stimulated NK cell LAMP‐1 and LAMP‐2 expression

The results for both absolute stimulated cell counts of NK LAMP‐1/LAMP‐2 expression and percentage positive for the receptors are shown in Table 2 and Figure 1, respectively. At Week 0, there were no between‐group differences in the absolute numbers of stimulated NK cells expressing LAMP‐1/LAMP‐2. However, at Week 12, UC was significantly lower than GE (*P *=* *0.013) and IE (*P *=* *0.025), while there was a trend to significance between UC and CTL (*P *=* *0.094). Pre‐ to postintervention, both GE and IE showed increased counts of NK LAMP‐1/LAMP‐2 (*P *=* *0.044 ES: 0.6; *P *=* *0.012 ES: 1.2), respectively. With percentage of NK cells positive for LAMP‐1/LAMP‐2, there were no between‐group differences at Week 0, but at Week 12 both CTL and UC were significantly lower than GE (*P *=* *0.017 and *P *=* *0.007, respectively); UC was also lower than IE (*P *=* *0.047). Pre‐ to postintervention, the percent positive of LAMP‐1/LAMP‐2 for both GE and IE significantly increased (*P *=* *0.021 ES: 0.8; *P *=* *0.049 ES: 0.5, respectively).

## Discussion

The primary aims of this study were to investigate the effects of GE and IE on cytotoxic T‐lymphocyte activation and NK cell degranulation in individuals with CFS/ME, compared to UC and non‐CFS/ME,. This is the first CFS/ME exercise training study that has compared different types of exercise program delivery and the effects on CD8^+^ and NK cell activation. Our most important findings were that irrespective of GE or IE training, postintervention cell counts and percent positive of CD3^‐^CD56^+^CD16^+^LAMP‐1/LAMP‐2 receptors increased significantly, suggesting that degranulation of NK cells was enhanced with exercise in CFS/ME patients. However, neither GE nor IE had a significant effect on CD8^+^CD38^+^ expression.

Acute exercise bout effects on NK cell function include a substantial and immediate postexercise increase in circulating cell numbers, due to a catecholamine‐induced egress from bone marrow and shear stress from increased blood flow (Bigley et al. 2015). However, the lymphocytosis is markedly greater in healthy individuals than in those with existing viral infections such as cytomegalovirus (CMV) (Bigley et al. 2015). The effects of moderate‐intensity training with previously sedentary participants include increased NK cell activation and cytotoxicity (Woods et al. 1999); furthermore, high volume aerobic exercise training increases CD107a expression (Moro‐Garcia et al. 2014), and individuals with high aerobic capacity have greater NK cell cytotoxic activity than less fit or sedentary individuals (Nieman et al. 1993; Walsh et al. 2011). It is certainly possible that our GE and IE CFS/ME groups developed an exercise adaptation, with either increased NK cell numbers overall or a greater percentage of the more cytotoxic CD56^dim^ in circulation (Idorn and Hojman 2016). CD56^dim^ are stored mainly within the spleen and vascular bed so increased blood flow, shear stress, and adrenergic signaling with subsequent down‐regulation of adhesion molecules (Walsh et al. 2011) may increase circulating numbers with enhanced LAMP receptor expression (Idorn and Hojman 2016; Moro‐Garcia et al. 2014). The CD56^dim^ subgroup have a greater expression of *ß*_1_ and *ß*_2_ adrenergic receptors than CD56^bright^, and therefore may be more sensitive to increased catecholamines during exercise, although the sustained increase we found after 12 weeks of training is not fully explained by this mechanism. It is also possible that exercise causes a phenotype shift from CD56^bright^ to CD56^dim^, as the former are regarded as precursor cells (Idorn and Hojman 2016; Walsh et al. 2011), which would increase the overall numbers of CD56^dim^. This concept needs further investigation.

LAMP‐1 and LAMP‐2 surface expression are positively correlated with cytotoxic granule content, and help protect NK cells during the degranulation process, avoiding apoptosis (Cohnen et al. 2013), so exercise‐associated increases in LAMP‐1/LAMP‐2 expression would enhance immune surveillance and reduce the risk of infections in CFS/ME individuals. Moro‐Garcia et al. (2014)suggested that an exercise‐induced increase in NK cell counts and functional capacity might represent a compensatory mechanism for the functional defects of CD8^+^ lymphocytes in individuals with viral infections such as CMV. Exercise‐associated intracellular signaling and gene expression may be implicated in increased receptor expression. Fairey et al. (2005) reported increased NK cell cytotoxicity in breast cancer patients after a 15‐week exercise program, and suggested that transcription of NK activation receptor genes was enhanced with exercise. A review by Bloch et al. (2013) also reported that physical activity increased NK cell cytotoxicity and proliferation, and reduced tumor cell invasions, in cancer patients. Recently, Zimmer et al. (2014, 2015) found increases in histone acetylation and DNA methylation with endurance exercise, and found an up‐regulation of gene transcription for NK cell activation receptors. Acute exercise bouts can increase the expression of the killer‐cell immunoglobulin‐like receptor (KIR) gene (Maltseva et al. 2011; Radom‐Aizik et al. 2013) and the FasL gene (Radom‐Aizik et al. 2013), so it is possible that intracellular signaling pathways for LAMP‐1/LAMP‐2 are also enhanced with regular exercise. Several studies have found that the mitogen‐activated protein kinase (MAPK/MEK1/2) pathway, including the extracellular signal‐regulated kinases (ERK1/2) and p38 signaling molecules, was impaired in CFS/ME and furthermore, that NK cells were significantly lower in cytoplasmic calcium than non‐CFS/ME, thus reducing intracellular signaling capacity through the ZAP‐70/Lck/phosphoinositide 3‐kinase (PI3K) pathway (Chacko et al. 2016; Huth et al. 2016; Nguyen et al. 2016).

Exercise training may improve intracellular signaling through (1) increased phosphorylation within the MAPK/protein kinase B (Akt) and Syk/ZAP‐70 pathways (Egan et al. 2010); and (2) increased tyrosine kinase phosphorylation, calcium flux/signaling within the PI3K pathway, as the release of cytotoxic granules is calcium‐dependent (Chacko et al. 2016; Maher et al. 2005). Furthermore, there is a critical threshold for signaling of MAPK and ERK for NK cells to produce an effector cell response, therefore increased phosphorylation and calcium flux may well enhance such responses (Chacko et al. 2016). There is ample evidence that both aerobic exercise and resistance training increases AMP‐activated protein kinase (AMPK), MAPK/ERK and p38 phosphorylation, and calcium/calmodulin signaling cascades in skeletal muscle cells (Egan et al. 2010; Williamson et al. 2003), and similar mechanisms have been proposed for exercise‐induced increases in T‐helper cell function in CFS/ME and some cancers (Bloch et al. 2013; Broadbent and Coutts 2016). It has been also shown that some specific ion channels in NK cells, which assist with calcium signaling, lytic granule fusion, the release of perforin, and mitochondrial function, are variant in CFS/ME patients (Marshall‐Gradisnik et al. 2016; Nguyen et al. 2016), and it is possible that exercise training has a positive effect on channel signaling.

We found that the CTL group had a significantly higher absolute count of stimulated CD3^+^CD8^+^ lymphocytes preintervention compared to CFS/ME groups, suggesting an impaired cytotoxic T‐cell response in the CFS/ME groups (Brenu et al. 2010). Previous studies have reported no differences in absolute numbers of circulating, unstimulated CD3^+^CD8^+^ lymphocytes between CFS/ME and non‐CFS/ME groups (Brenu et al. 2010; Broadbent and Coutts 2016; Curriu et al. 2013; Robertson et al. 2005) but investigations of CD8^+^ activation and function are less clear, due to different target receptors assessed and varying laboratory methodologies. The effects of moderate‐intensity exercise training on CD8^+^ lymphocyte function, as opposed to cell counts alone, in healthy individuals and athletes, have not been deeply investigated. Acute bouts of exercise, often incremental or high‐intensity, result in lymphocytosis up to 1 h post‐exercise, with possible reductions in cytotoxic function (Walsh et al. 2011). Acute exercise also increases redeployment of CD8^+^ T‐cell subsets in people with CMV (Spielmann et al. 2014).

The CTL group showed a significantly lower count of CD3^+^CD8^+^CD38^+^ than UC and IE. CD38^+^ has a multifunctional role as an “activation marker” and as a multifunctional enzyme that moderates intracellular NAD^+^, cADPR, and calcium flux, possibly with a potential to regulate or enhance the T‐cytotoxic and other innate immune responses during inflammation, and to modulate lymphocyte apoptosis (Hamblin 2003; Quarona et al. 2013). Elevated CD38^+^ expression is associated with the progression of EBV, HIV, and some cancers (Quarona et al. 2013; Zidovec Lepej et al. 2003). However, CD38 expression has been reported in very few CFS/ME studies. Robertson et al. (2005) and Zhang et al. (1999) found that CFS/ME patients had increased numbers of activated cytotoxic T cells co‐expressing CD8 with CD38, whereas Curriu et al. (2013) reported CFS/ME individuals had lower numbers of CD8^+^CD38^+^ but with higher expression of the anergic marker CD5. The proposed explanations for these findings were that more symptomatic CFS/ME patients with current inflammation, might exhibit greater CD8^+^CD38^+^ activation (Robertson et al. 2005) or that CD8^+^ hypo‐responsiveness was due to continual antigen exposure during ongoing inflammation (Curriu et al. 2013).

There is no literature reporting the effects of exercise on CD8^+^CD38^+^ expression. We found modest but nonsignificant postintervention decreases in CD38 expression in the GE and IE CFS/ME groups, which reduced CD38 to much the same level as in the CTL group. Although our sample size was small, we could suggest that both modalities of low‐moderate intensity exercise (1) had an anti‐inflammatory effect or (2) enhanced the immunomodulatory activity of the receptor (Quarona et al. 2013). The action of CD38^+^ in cell transmembrane signaling can lead to the production of interleukin‐1 (IL‐1), TNF‐*α*, and GM‐CSF (i.e., proinflammatory responses); if exercise training lowered CD8^+^CD38^+^ expression, it might also reduce the concentration of these cytokines. This would be some evidence toward low‐intensity exercise as a mechanism to decrease inflammation in CFS/ME, and possibly other immunological conditions such as cancer (Erlandson et al. 2013; Evans et al. 2015; Fairey et al. 2005; Zimmer et al. 2014). Further investigation into the actions of this ectoenzyme within CFS/ME and possible interactions with exercise is warranted.

We found that 12 weeks of GE and IE increased aerobic capacity without exacerbating fatigue or other CFS/ME symptoms. While the GE finding was consistent with other studies (Gordon et al. 2010; Wallman et al. 2004), IE was equally effective, with participants reporting this modality to be less tiring overall (Broadbent and Coutts 2016). This is an important finding for CFS/ME individuals who are symptom‐limited for exercise participation; IE may be a more manageable modality compared to GE.

### Limitations of the study

We suggest some caution when interpreting the results because the cohort size of the pilot study was small and gender data were also pooled in each group. However, we reported moderate to large effect sizes for the most significant results and further more robust investigation of both IE and GE for CFS/ME individuals is recommended, focusing on both immunological and clinical outcomes.

## Conclusions

Twelve weeks of both GE and IE with CFS/ME individuals significantly increased expression of NK cell LAMP‐1/LAMP‐2, and enhanced NK cell degranulation/activation is an important clinical finding for improved immune function. GE and IE may also lower CD8^+^CD38^+^ expression, indicating reduced inflammatory responses in both exercise groups. Furthermore, both GE and IE improved exercise capacity without worsening CFS/ME symptoms, and larger and longer duration trials of these exercise modalities and outcome measures for CFS/ME are warranted.

## Conflict of Interest

The authors declare no conflict of interest.
